# Comparative Analysis of Retrobulbar Blood Flow in Symmetric and Asymmetric Keratoconus Patients

**DOI:** 10.3390/jcm14165717

**Published:** 2025-08-12

**Authors:** Fatma Huriye Kısa, Hüseyin Findik, Feyzahan Uzun, Muhammet Kaim, Merve Solak, Mehmet Gökhan Aslan

**Affiliations:** 1 Department of Ophthalmology, School of Medicine, Recep Tayyip Erdogan University, 53100 Rize, Turkey; fatmahuriye.kisa@erdogan.edu.tr (F.H.K.); feyzahan.ekici@erdogan.edu.tr (F.U.); muhammet.kaim@erdogan.edu.tr (M.K.); mehmetgokhan.aslan@erdogan.edu.tr (M.G.A.); 2 Department of Radiology, School of Medicine, Recep Tayyip Erdogan University, 53100 Rize, Turkey; merve_solak23@erdogan.edu.tr

**Keywords:** keratoconus, retrobulbar hemodynamics, color doppler ultrasonography, ophthalmic artery, central retinal artery, posterior ciliary artery

## Abstract

**Background/Objectives:** Keratoconus is a progressive corneal disorder characterized by thinning and conical protrusion of the cornea, resulting in visual impairment. This study aimed to evaluate retrobulbar blood flow characteristics in patients with symmetric and asymmetric keratoconus and to compare these parameters with those of healthy individuals. **Methods:** Participants aged 18–40 years were recruited and categorized into three groups: symmetric keratoconus, asymmetric keratoconus, and healthy controls. Color Doppler ultrasonography was used to measure the pulsatility index (PI) and resistive index (RI) of the ophthalmic artery (OA), central retinal artery (CRA), and posterior ciliary artery (PCA). Retrobulbar hemodynamic parameters were analyzed and compared across groups using appropriate statistical methods. **Results:** The ophthalmic artery PI (oaPI) and central retinal artery PI (craPI) were significantly elevated in both symmetric keratoconus patients and the affected eyes of asymmetric keratoconus patients compared to the control group (*p* < 0.05). In contrast, the oaPI in the unaffected eyes of asymmetric keratoconus patients was significantly lower than that of controls (*p* < 0.05). **Conclusions:** The elevated oaPI and craPI values observed in keratoconus patients suggest that the disease may involve not only corneal structural abnormalities but also alterations in ocular blood flow. These findings may imply a potential vascular component in keratoconus pathophysiology.

## 1. Introduction

Keratoconus (KC) is a progressive, bilateral, and often asymmetric corneal disorder characterized by stromal thinning, paracentral or central corneal protrusion, and irregular astigmatism. Typically manifesting during adolescence, the disease may progress until the fourth decade of life, leading to varying degrees of visual impairment and a significant reduction in quality of life [1].

Although KC has traditionally been regarded as a non-inflammatory condition [2], growing evidence suggests that inflammatory mechanisms may contribute to its pathogenesis [3,4]. The exact etiology remains elusive, but it is widely accepted that both genetic and environmental factors play a role. Mechanical trauma, particularly vigorous eye rubbing, has been identified as a significant risk factor [5], and behavioral patterns such as sleeping position (e.g., prone or lateral decubitus) have been implicated in the development of unilateral or asymmetric disease presentations [6].

KC presents with varying clinical patterns, most notably symmetric and asymmetric forms. Symmetric KC typically involves bilateral corneal changes of similar severity, suggesting a more generalized or systemic predisposition, potentially influenced by inherent biomechanical weakness or vascular components. In contrast, asymmetric KC is characterized by a significant difference in disease severity between the two eyes, often attributed to localized environmental or behavioral factors such as habitual eye rubbing, preferential sleeping position, or ocular dominance [7]. These differing clinical expressions may reflect distinct underlying pathophysiological mechanisms. Investigating vascular characteristics in these subtypes may, therefore, offer new insights into whether retrobulbar hemodynamics play a contributory role in either localized or systemic progression of the disease.

Emerging evidence suggests that ocular blood flow may influence the metabolic and structural integrity of the cornea [8]. Disruption of normal perfusion to the anterior segment, including the pericorneal and retrobulbar circulation, could potentially contribute to corneal thinning and ectasia through mechanisms such as tissue hypoxia, oxidative stress, or impaired extracellular matrix remodeling [9]. Moreover, asymmetrical ocular blood flow may help explain the frequent asymmetry observed in KC patients. Therefore, assessing retrobulbar hemodynamics may provide new insights into the pathophysiology of KC and its variable clinical course [10].

Color Doppler ultrasonography (CDU) is a non-invasive imaging modality that enables the evaluation of blood flow velocity and resistance in orbital vessels such as the ophthalmic artery (OA), central retinal artery (CRA), and posterior ciliary artery (PCA). Alterations in retrobulbar hemodynamics have been documented in a variety of ocular diseases, including glaucoma, optic neuropathies, age-related macular degeneration, and diabetic retinopathy [11]. Such hemodynamic parameters, already linked to disease severity in several ocular pathologies, may similarly offer insight into the vascular dynamics underlying KC.

Despite these associations, studies examining the role of retrobulbar circulation in KC are limited. Given the disease’s bilateral yet often asymmetric progression, investigating retrobulbar hemodynamics may help elucidate underlying mechanisms. In this study, retrobulbar blood flow parameters were compared among patients with symmetric and asymmetric KC and healthy individuals to explore possible vascular contributions to the disease’s pathogenesis and progression.

## 2. Materials and Methods

### 2.1. Study Design and Ethical Considerations

This prospective, observational study was conducted in accordance with the principles of the Declaration of Helsinki. The study protocol was approved by the institutional ethics committee. All participants were informed about the study’s objectives, and written informed consent was obtained from each participant prior to enrollment.

### 2.2. Study Population

The study included individuals aged between 18 and 40 years, comprising both patients diagnosed with KC and healthy controls within the same age range. All participants self-identified as ethnically Turkish, reflecting the homogeneous demographic structure of the study population. The diagnosis of KC was established according to the Amsler–Krumeich classification [12]. Patients showing a difference in two or more stages between their eyes, or those with KC in only one eye, were classified as having asymmetric KC. In contrast, patients with less than a two-stage difference between the eyes were considered to have symmetric KC. Age-matched healthy individuals with no signs of KC or other ocular diseases were included as the control group.

### 2.3. Inclusion and Exclusion Criteria

Participants were eligible if they were between 18 and 40 years of age and had a confirmed diagnosis of KC based on the Amsler–Krumeich criteria. Individuals were excluded if they had a history of ocular surgery, trauma, uveitis, other corneal pathologies, eyelid anomalies, or blepharitis. The use of systemic medications was also considered an exclusion criterion. Furthermore, individuals with systemic or autoimmune conditions, including pregnancy, breastfeeding, Sjögren’s syndrome, or rheumatoid arthritis, were not included in the study.

### 2.4. Sample Size and Group Classification

A total of 116 participants were included in the study. Among them, 39 patients (78 eyes) were classified in the asymmetric KC group, 37 patients (74 eyes) in the symmetric KC group, and 40 healthy individuals (80 eyes) comprised the control group.

### 2.5. Ophthalmological Examination and Topographic Evaluation

All participants underwent a detailed ophthalmologic examination followed by corneal topography using the Sirius topography device (Costruzione Strumenti Oftalmici, Florence, Italy). The following parameters were recorded for each participant: maximum keratometry (Kmax), average keratometry (SimK), minimum central corneal thickness (MCCT), anterior and posterior vertex refractive indices (Kvf and Kvb, respectively), corneal volume (CV), and cylindrical diopter (CylD). The staging of KC was reassessed and confirmed using the Amsler–Krumeich classification based on the obtained topographic data.

### 2.6. Color Doppler Ultrasonography

Retrobulbar blood flow was evaluated in all participants using color Doppler ultrasonography (CDU), performed with a Samsung V8 ultrasound device (Samsung Medison Co., Seoul, Republic of Korea). All examinations were conducted by a single experienced radiologist who was blind to the clinical status of the participants in order to minimize measurement bias and ensure procedural consistency.

Before the procedure, each patient received a detailed explanation of the examination technique. Patients were then instructed to lie in a supine position with their head elevated at approximately 30 degrees. The eyes were gently closed, and patients were asked to maintain a steady gaze in the primary position, looking straight ahead, under closed eyelids, to minimize ocular motion. The ultrasound probe was positioned horizontally over the upper eyelid to acquire an axial view of the orbit. To avoid external pressure that could affect blood flow dynamics, minimal transducer pressure was applied to the globe. An adequate amount of coupling gel was used to ensure optimal acoustic contact without direct compression of the eyeball.

**Figure 1:**
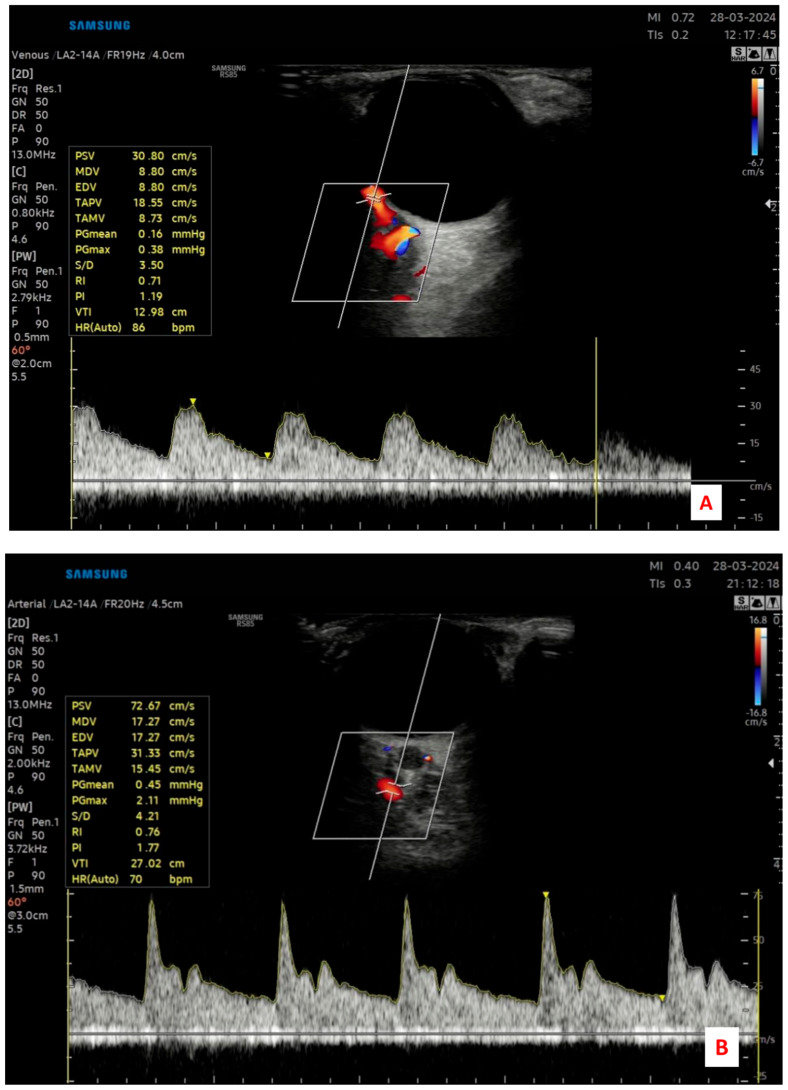

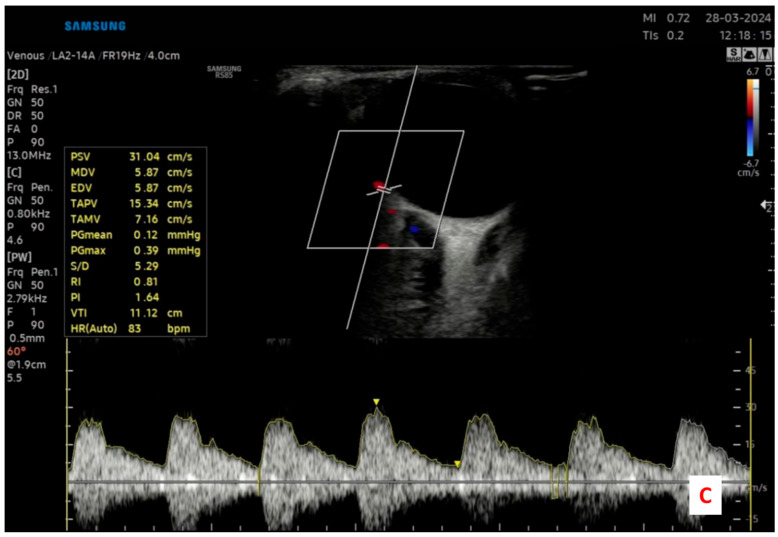
Representative color Doppler ultrasonography images demonstrating the retrobulbar blood flow assessment in a study participant. (**A**) Central retinal artery (CRA) identified within the optic nerve sheath; (**B**) Ophthalmic artery (OA) measured at the orbital apex; and (**C**) Posterior ciliary artery (PCA) evaluated near the optic nerve entry site.

Measurements were taken from the ophthalmic artery (OA), central retinal artery (CRA), and posterior ciliary artery (PCA). For each vessel, the pulsatility index (PI) and resistive index (RI) were automatically calculated by the device’s built-in software, using waveform analysis. Care was taken to maintain the angle of insonation at ≤60° to optimize Doppler signal accuracy and avoid angular artifacts. The sample volume was adjusted to the vessel size, and the gate was placed in the center of the vessel lumen during measurement. Each measurement was obtained when a clear and stable Doppler waveform was visible, and three consecutive cardiac cycles were recorded for consistency (Figure 1).

All measurements were performed bilaterally for each participant under the same standardized conditions, and the CDU protocol was applied identically across all groups to ensure reproducibility and reduce inter-individual variability.

### 2.7. Statistical Analysis

Data analysis was performed using IBM SPSS Statistics for Windows, Version 29.0 (IBM Corp., Armonk, NY, USA). Categorical variables were presented as frequencies and percentages. Continuous variables were assessed for normality using the Kolmogorov–Smirnov test. Variables with a normal distribution were presented as means ± standard deviations (SD), while non-normally distributed variables were expressed as medians along with interquartile ranges (IQR; Q1–Q3) and minimum–maximum values.

Comparisons between two groups were performed using the Independent Samples T-test for normally distributed variables, or the Mann–Whitney U test for non-normally distributed variables. For comparisons involving three or more groups, One-Way ANOVA or the Kruskal–Wallis H test was employed, depending on the distribution characteristics. Bonferroni correction was applied for post hoc analyses. A *p*-value of less than 0.05 was considered statistically significant.

## 3. Results

A total of 116 individuals were included in the study. The demographic characteristics of the participants are summarized in Table 1. The participants’ ages ranged from 19 to 40 years, with a mean and median age of 29 years. Of the total cohort, 37.9% (n = 44) were male and 62.1% (n = 72) were female. Regarding group distribution, 31.9% (n = 37) of the participants were classified as symmetric KC patients, 33.6% (n = 39) as asymmetric KC patients, and 34.5% (n = 40) comprised the control group.

For the symmetric KC and control groups, the mean of the right and left eye measurements was calculated and used in the analysis. In contrast, for the asymmetric KC group, the eyes with and without KC were analyzed separately.

The distribution of retrobulbar blood flow and corneal topographic parameters in the eyes of patients with asymmetric KC, based on the presence or absence of disease, is presented in Table 2. Analysis of the data revealed a significant increase in the ophthalmic artery pulsatility index (oaPI) in eyes affected by KC compared to the unaffected fellow eyes. Additionally, all topographic parameters demonstrated statistically significant differences between the affected and unaffected eyes (*p* < 0.05).

The distribution of retrobulbar blood flow and corneal topographic parameters in symmetric KC patients and healthy control subjects is presented in Table 3. Analysis of the data revealed statistically significant differences between the two groups in the ophthalmic artery pulsatility index (oaPI) and the central retinal artery pulsatility index (craPI) among the vascular parameters. Additionally, significant differences were observed in several topographic measurements, including Kvf, Kvb, MCCT, CV, CylD, SimK1, SimK2, hoa, and coma values (*p* < 0.05).

The distribution of retrobulbar blood flow and corneal topographic parameters among symmetric KC patients, asymmetric KC patients (affected eyes only), and healthy control subjects is presented in Table 4. Analysis of the data revealed that the ophthalmic artery pulsatility index (oaPI) was significantly higher in the symmetric KC group compared to the control group. Statistically significant differences were also observed in topographic parameters, including Kvf, Kvb, MCCT, CV, CylD, SimK1, SimK2, hoa, and coma values (*p* < 0.05). To determine the source of these differences, a post hoc Bonferroni test was performed, which indicated that the significant variations in all topographic measurements were attributable to differences between the control group and both the symmetric KC group and the KC-affected eyes of the asymmetric group.

The distribution of retrobulbar blood flow and corneal topographic parameters in the non-KC eyes of asymmetric patients, the eyes of symmetric KC patients, and healthy control subjects is presented in Table 5. Statistical analysis revealed significant differences among the groups in the ophthalmic artery resistive index (oaRI) and pulsatility index (oaPI), as well as in several topographic parameters, including Kvf, Kvb, MCCT, CV, CylD, SimK1, SimK2, hoa, coma, and spherical aberration (SA) (*p* < 0.05). To determine the specific group differences contributing to these findings, a post hoc Bonferroni test was conducted.

## 4. Discussion

In this study, we evaluated retrobulbar blood flow in patients with symmetric and asymmetric KC compared to healthy controls using color Doppler ultrasonography (CDU). Our results demonstrated significantly elevated pulsatility index (PI) values in the ophthalmic artery (oaPI) and central retinal artery (craPI) in eyes affected by KC. These findings suggest that vascular alterations in retrobulbar circulation may contribute to the pathophysiology of KC. Additionally, significant differences in corneal topographic parameters between KC groups and controls further support the association between structural and circulatory changes in the disease process.

CDU is a reliable, non-invasive, and reproducible method for evaluating the hemodynamic characteristics of retrobulbar vessels [13]. By measuring parameters such as the pulsatility index (PI) and resistive index (RI) in vessels including the ophthalmic artery (OA), central retinal artery (CRA), and posterior ciliary artery (PCA), CDU has contributed significantly to our understanding of the pathophysiology of various ocular and systemic diseases [14].

Comparable alterations in retrobulbar blood flow parameters have been reported in other ocular conditions. For example, Yu et al. observed increased RI values alongside reduced peak systolic velocity (PSV) and end-diastolic velocity (EDV) in the OA, CRA, and PCA in patients with pathological myopia [15]. However, that study did not assess the PI parameter. Likewise, Aydın et al. reported significantly elevated oaRI values in amblyopic eyes compared to healthy controls in patients with anisometropic amblyopia, although the increase in oaPI did not reach statistical significance [16].

Our study adds to this body of evidence by demonstrating that both oaPI and craPI are elevated in KC eyes, regardless of disease symmetry. These findings highlight the potential role of retrobulbar vascular alterations in the pathogenesis of KC.

Although KC has long been considered a non-inflammatory disorder, accumulating evidence suggests that inflammation may play a role in its development [17]. The observed increase in PI values in our study may reflect inflammation-mediated changes in retrobulbar vasculature. Supporting this hypothesis, previous research in patients with rheumatoid arthritis—a systemic inflammatory disease—has demonstrated significantly elevated PI and RI values in the OA, CRA, and PCA [18]. These parallels suggest that retrobulbar hemodynamic alterations in KC may also be linked to low-grade chronic inflammation.

Furthermore, the impact of KC may not be limited to the cornea. Aksoy Aydemir et al. reported significant thinning of the retinal nerve fiber layer (RNFL) in KC patients [19]. Similarly, Grundinska et al. demonstrated significant correlations between retrobulbar blood flow parameters and the thickness of the RNFL and ganglion cell layer in myopic eyes [20]. These findings reinforce the idea that KC may be part of a broader pathophysiological process involving both anterior and posterior segment structures, including retinal and retrobulbar components. The elevated oaPI and craPI values observed in our study further support this broader disease model.

Altered retrobulbar hemodynamics have been implicated in various other ocular diseases as well. For instance, in thyroid eye disease, Cemşidyan-Tahrani et al. reported significant changes in the end-diastolic velocity (EDV) and resistive index (RI) values of the ophthalmic artery (OA), associating these alterations with disease activity [21]. Similarly, in prediabetic individuals, a study by Sirkeci et al. demonstrated marked elevations in the RI values of all retrobulbar vessels, suggesting that such changes may indicate early stages of retinopathy and systemic microangiopathy [22]. In the context of central serous chorioretinopathy, Hamidi et al. [23] found elevated PI and RI values, supporting the hypothesis that vasospasm may contribute to the pathophysiology of the disease. These examples from the literature support the notion that increased oaPI and craPI values, as observed in our study, may reflect vascular dysregulation and contribute to the pathogenesis of KC.

Although this study primarily focused on PI as a sensitive marker of retrobulbar hemodynamic alterations, RI values were also evaluated across all vessel groups. Interestingly, RI did not show significant differences between KC patients and controls in most comparisons. This finding does not necessarily imply the absence of vascular involvement, as RI is influenced not only by distal vascular resistance but also by vessel wall compliance and flow dynamics, making its interpretation more complex in early or functional disease states. In contrast, PI has been shown to be more sensitive to subtle changes in vascular impedance and compliance, particularly in subclinical or early pathological processes [13]. Previous studies in systemic and ocular vascular conditions, such as prediabetes and hypertension, have reported that PI alterations may precede changes in RI, reflecting an earlier stage of microvascular dysregulation [24,25]. Therefore, the lack of significant RI differences in our study may reflect the relatively early or functional nature of retrobulbar changes in KC, rather than an absence of hemodynamic involvement.

Interestingly, we also observed that the oaPI was significantly lower in the unaffected eyes of asymmetric KC patients compared to both control and symmetric KC groups. Although unexpected, this finding may reflect compensatory or subclinical vascular mechanisms. Evidence from vascular research demonstrates that ocular circulation can undergo adaptive responses to altered hemodynamics [26,27]. Theoretically, the unaffected eye in asymmetric KC may exhibit lower oaPI as an early, adaptive vasoregulatory response to maintain corneal homeostasis before topographic manifestations develop. Alternatively, this reduction may represent an early phase of vascular dysregulation that precedes the elevated oaPI seen in overtly affected eyes. These asymmetric vascular changes could thus mirror the clinical asymmetry often seen in KC. Longitudinal studies with serial vascular imaging and corneal monitoring are required to clarify whether such hemodynamic alterations may serve as early indicators of disease progression in the contralateral eye.

This study has several limitations. First, peak systolic velocity (PSV) and end-diastolic velocity (EDV) values were not included in the evaluation of retrobulbar blood flow parameters. Assessment of these indices could provide a more comprehensive understanding of the hemodynamic characteristics of retrobulbar vessels. Second, optical coherence tomography angiography (OCTA) and RNFL measurements were not performed. Including these parameters could further elucidate the relationship between retrobulbar circulation and retinal structural changes. Third, while the present study focused on comparing retrobulbar blood flow characteristics between symmetric, asymmetric, and control groups, it did not stratify patients based on the severity of KC within those groups. A subgroup analysis based on disease stage was not performed due to limitations in sample size and statistical power. Additionally, the absence of detailed data on refractive status, axial length, and family history of high myopia may have introduced potential confounding effects on retrobulbar blood flow measurements. Future studies should aim to address these limitations to enhance the depth and clinical relevance of the findings.

In conclusion, this study demonstrated that retrobulbar blood flow parameters—specifically the ophthalmic artery pulsatility index (oaPI) and central retinal artery pulsatility index (craPI)—are significantly elevated in patients with KC compared to healthy individuals. These findings suggest that KC may not be limited to corneal structural abnormalities but may also involve broader pathophysiological changes affecting retrobulbar circulation. As the first study to explore the relationship between KC and retrobulbar hemodynamics, our results provide novel insights into the disease process. The identification of altered retrobulbar hemodynamics in KC patients, particularly the elevated PI values in the ophthalmic and central retinal arteries, raises important considerations for clinical practice. These parameters may have potential as non-invasive hemodynamic biomarkers for the early detection of subclinical vascular involvement, monitoring disease progression, or assessing asymmetry in KC. In particular, eyes that are clinically unaffected but demonstrate vascular deviations could warrant closer follow-up, especially in asymmetric cases. Moreover, if future longitudinal studies confirm a link between retrobulbar blood flow alterations and KC progression, these findings may pave the way for novel therapeutic strategies targeting ocular perfusion and vascular health. Such approaches could complement existing corneal-focused treatments and contribute to a more comprehensive management framework for KC, particularly in its early or asymmetric stages.

## Figures and Tables

**Table 1:** Demographic characteristics of the participants.

Variables (N = 116)	N (%)	Mean ± SD	Median (Min–Max) IQR (Q1–Q3)
Gender			
Female	72 (62.1%)		
Male	44 (37.9%)		
Age (years)		29 ± 5	29 (19–40) (23–32)
Group			
Symmetric	37 (31.9%)		
Asymmetric	39 (33.6%)		
Control	40 (34.5%)		

**Table 2:** Distribution of Blood Flow and Topographic Measurements According to the Presence or Absence of KC in the Eyes of Asymmetric Patients.

Variables	Asymmetric KC Present	Asymmetric KC Absent	*p* Value
**oaRI**	0.77 (0.54–0.96)	0.74 (0.6–9.6)	0.122 **
**Median (Min–Max)**			
**oaPI**	**1.99 ± 1.01**	**1.73 ± 0.51**	0.045 *
**Mean ± SD**			
**craRI**			0.800 **
**Median (Min–Max)**	0.76 (0.5–0.94)	0.75 (0.48–0.92)	
**IQR (Q1–Q3)**	(0.65–0.81)	(0.69–0.79)	
**craPI**			0.697 **
**Median (Min–Max)**	1.48 (0.55–2.56)	1.38 (0.68–2.73)	
**IQR (Q1** **–** **Q3)**	(1.13–1.87)	(1.14–1.93)	
**pcaRI**			0.996 **
**Median (Min–Max)**	0.69 (0.53–0.94)	0.70 (0.52–6.4)	
**IQR (Q1–Q3)**	(0.65–0.77)	(0.65–0.76)	
**pcaPI**			0.887 **
**Median (Min–Max)**	1.24 (0.52–2.87)	1.27 (0.75–2.55)	
**IQR (Q1–Q3)**	(1.14–1.73)	(1.13–1.60)	
**Kvf**			<0.001 **
**Median (Min–Max)**	**28 (6–57)**	**9 (3–42)**	
**IQR (Q1–Q3)**	**(22.00–40.0)**	**(5.00–14.0)**	
**Kvb**			<0.001 **
**Median (Min–Max)**	**67 (13–129)**	**25 (7–102)**	
**IQR (Q1–Q3)**	**(52.00–94.0)**	**(14.0–33.0)**	
**MCCT**	**460.67 ± 36.96**	**492.97 ± 34.54**	<0.001 *
**Mean ± SD**			
**CV**			<0.001 **
**Median (Min–Max)**	**2.81 (0.29–5.86)**	**0.88 (0.12–3.46)**	
**IQR (Q1–Q3)**	**(2.22–3.81)**	**(0.51–1.34)**	
**CylD**			<0.001 **
**Median (Min–Max)**	**−3.1 (−9.61–−0.23)**	**−1.28 (−4.98–−0.2)**	
**IQR (Q1–Q3)**	**(−4.21–−1.63)**	**(−1.85–−0.69)**	
**SimK1**	**45.38 ± 2.37**	**43.69 ± 2.07**	0.001 *
**Mean ± SD**			
**SimK2**	**48.22 ± 2.83**	**45.26 ± 2.41**	<0.001 *
**Mean ± SD**			
**Hoa**			<0.001 **
**Median (Min–Max)**	**1.39 (0.41–2.94)**	**0.49 (0.15–2.41)**	
**IQR (Q1–Q3)**	**(0.96–1.90)**	**(0.32–0.69)**	
**Coma**			<0.001 **
**Median (Min–Max)**	**1.18 (0.32–2.49)**	**0.36 (0.04–1.75)**	
**IQR (Q1** **–** **Q3)**	**(0.77–1.60)**	**(0.12–0.49)**	
**Sa**			<0.001 **
**Median (Min–Max)**	**0.18 (0–1.12)**	**0.08 (0–1.04)**	
**IQR (Q1** **–** **Q3)**	**(0.09–0.25)**	**(0.03–0.15)**	

* Independent Sample *T*-test, ** Mann–Whitney U Test, bold: statistically significant, KC: Keratoconus, oaRI: Ophthalmic Artery Resistive Index, oaPI: Ophthalmic Artery Pulsatility Index, craRI: Central Retinal Artery Resistive Index, craPI: Central Retinal Artery Pulsatility Index, pcaRI: Short Posterior Ciliary Artery Resistive Index, pcaPI: Short Posterior Ciliary Artery Pulsatility Index, Kvf: Anterior vertex refractive index, Kvb: Posterior vertex refractive indices index, MCCT: Minimum central corneal thickness, CV: Corneal volume, SimK1, SimK2 = Simulated Keratometry Readings (D), hoa, coma, sa: higher-order aberrations, and SD: Standart deviation.

**Table 3:** Comparison of Variables Between Control and Symmetric Groups.

Variable		Control Group	Symmetric KC Group	*p* Value
**oaRI**	Median (Min–Max)	0.78 (0.64–0.89)	0.78 (0.61–0.87)	0.740
	IQR (Q1–Q3)	(0.75–0.81)	(0.75–0.82)	
**oaPI**	Median (Min–Max)	**1.8 (1.02–3.14)**	**2 (1.26–2.77)**	**0.049**
	IQR (Q1–Q3)	**(1.50–2.02)**	**(1.58–2.26)**	
**craRI**	Mean ± SD	0.72 ± 0.08	0.74 ± 0.07	0.120
**craPI**	Median (Min–Max)	**1.41 (0.74–2.74)**	**1.6 (1.07–2.88)**	**0.026**
	IQR (Q1–Q3)	**(1.20–1.57)**	**(1.33–1.89)**	
**pcaRI**	Median (Min–Max)	0.72 (0.58–0.83)	0.74 (0.56–1.00)	0.308
	IQR (Q1–Q3)	(0.67–0.76)	(0.68–0.78)	
**pcaPI**	Median (Min–Max)	1.34 (0.91–2.41)	1.48 (0.89–5.05)	0.494
	IQR (Q1–Q3)	(1.19–1.64)	(1.24–1.71)	
**Kvf**	Median (Min–Max)	**4 (1.5–7)**	**25 (5.5–81.5)**	**<0.001**
	IQR (Q1–Q3)	**(3.50–5.25)**	**(16.0–31.0)**	
**Kvb**	Median (Min–Max)	**10 (6–16.5)**	**59 (9–168)**	**<0.001**
	IQR (Q1–Q3)	**(8.50–11.75)**	**(35.5–73.0)**	
**MCCT**	Mean ± SD	**531.95 ± 29.36**	**459.96 ± 47.04**	**<0.001**
**CV**	Median (Min–Max)	**0.04 (0–0.37)**	**2.46 (0–8.61)**	**<0.001**
	IQR (Q1–Q3)	**(0.00–0.12)**	**(1.60–3.72)**	
**CylD**	Median (Min–Max)	**−0.64 (−1.38–−0.13)**	**−2.33 (−7.42–−0.40)**	**<0.001**
	IQR (Q1–Q3)	**(−0.87–−0.39)**	**(−3.21–−1.73)**	
**SimK1**	Mean ± SD	**42.95 ± 1.53**	**44.72 ± 2.27**	**<0.001**
**SimK2**	Mean ± SD	**43.76 ± 1.58**	**47.10 ± 2.63**	**<0.001**
**hoa**	Median (Min–Max)	**0.4 (0.21–0.64)**	**1.04 (0.33–3.35)**	**<0.001**
	IQR (Q1–Q3)	**(0.32–0.50)**	**(0.74–1.73)**	
**coma**	Median (Min–Max)	**0.23 (0.06–0.53)**	**0.86 (0.22–3.05)**	**<0.001**
	IQR (Q1–Q3)	**(0.16–0.33)**	**(0.55–1.57)**	
**sa**	Median (Min–Max)	0.21 (0.07–12.04)	0.17 (0.02–0.68)	0.253
	IQR (Q1–Q3)	(0.14–0.27)	(0.11–0.26)	

Variables summarized as Mean ± SD were compared using the Independent Samples *T*-test. Variables summarized as Median (Min–Max) and IQR (Q1–Q3) were compared using the Mann–Whitney U Test. bold: statistically significant, KC: Keratoconus, oaRI: Ophthalmic Artery Resistive Index, oaPI: Ophthalmic Artery Pulsatility Index, craRI: Central Retinal Artery Resistive Index, craPI: Central Retinal Artery Pulsatility Index, pcaRI: Short Posterior Ciliary Artery Resistive Index, pcaPI: Short Posterior Ciliary Artery Pulsatility Index, Kvf: Anterior vertex refractive index, Kvb: Posterior vertex refractive indices index, MCCT: Minimum central corneal thickness, CV: Corneal volume, SimK1, SimK2 = Simulated Keratometry Readings (D), hoa, coma, sa: higher-order aberrations, and SD: Standart deviation.

**Table 4:** Distribution of Blood Flow and Topographic Measurements in Eyes with Keratoconus in Asymmetric Patients Compared to Symmetric and Control Groups.

Variables	Control ^1^	Symmetric ^2^	Asymmetric with KC ^3^	*p* Value	Post-HOC
**oaRI**	0.78	0.78	0.77	0.980 **	
**Median (Min-Max)**	(0.64–0.89)	(0.61–0.87)	(0.54–0.96)		
**IQR (Q1–Q3)**	(0.75–0.81)	(0.75–0.82)	(0.72–0.85)		
**oaPI**	**1.8**	**2**	**1.79**	0.039 **	**1** **–** **2**
**Median (Min-Max)**	**(1.02–3.14)**	**(1.26–2.77)**	**(0.76–7.23)**		
**IQR (Q1–Q3)**	**(1.50–2.02)**	**(1.58–2.26)**	**(1.51–2.23)**		
**craRI**	0.72 ± 0.08	0.74 ± 0.07	0.74 ± 0.10	0.287 *	
**Mean ± SD**					
**craPI**	1.41	1.6	1.48	0.096 **	
**Median (Min-Max)**	(0.74–2.74)	(1.07–2.88)	(0.55–2.56)		
**IQR (Q1–Q3)**	(1.20–1.57)	(1.33–1.89)	(1.13–1.87)		
**pcaRI**	0.72	0.74	0.69	0.428 **	
**Median (Min-Max)**	(0.58–0.83)	(0.56–1)	(0.53–0.94)		
**IQR (Q1–Q3)**	(0.67–0.76)	(0.68–0.78)	(0.65–0.77)		
**pcaPI**	1.34	1.48	1.24	0.355 **	
**Median (Min-Max)**	(0.91–2.41)	(0.89–5.05)	(0.52–2.87)		
**IQR (Q1–Q3)**	(1.19–1.64)	(1.24–1.71)	(1.14–1.73)		
**Kvf**	**4**	**25**	**28**	<0.001 **	**1** **–** **2; 1** **–** **3**
**Median (Min-Max)**	**(1.5–7)**	**(5.5–81.5)**	**(6–57)**		
**IQR (Q1–Q3)**	**(3.50–5.25)**	**(16.0–31.0)**	**(22.0–40.0)**		
**Kvb**	**10**	**59**	**67**	<0.001 **	**1** **–** **2; 1** **–** **3**
**Median (Min-Max)**	**(6–16.5)**	**(9–168)**	**(13–129)**		
**IQR (Q1–Q3)**	**(8.50–11.75)**	**(35.5–73.0)**	**(52.0–94.0)**		
**MCCT**				<0.001 *	**1** **–** **2; 1** **–** **3**
**Mean ± SD**	**531.95 ± 29.36**	**459.96 ± 47.04**	**460.67 ± 36.96**		
**Kmax**	**0.04**	**2.46**	**2.81**	<0.001 **	**1** **–** **2; 1** **–** **3**
**Median (Min-Max)**	**(0–0.37)**	**(0–8.61)**	**(0.29–5.86)**		
**IQR (Q1–Q3)**	**(0.0–0.12)**	**(1.60–3.72)**	**(2.22–3.81)**		
**CylD**	**−0.64**	**−2.33**	**−3.1**	<0.001 **	**1** **–** **2; 1** **–** **3**
**Median (Min-Max)**	**(−1.38–−0.13)**	**(−7.42–−0.4)**	**(−9.61–−0.23)**		
**IQR (Q1–Q3)**	**(−0.87–−0.39)**	**(−3.21–−1.73)**	**(−4.21–−1.63)**		
**SimK1**	**42.95 ± 1.53**	**44.72 ± 2.27**	**45.38 ± 2.37**	<0.001 *	**1** **–** **2; 1** **–** **3**
**Mean ± SD**					
**SimK2**	**43.76 ± 1.58**	**47.1 ± 2.63**	**48.22 ± 2.83**	<0.001 *	**1** **–** **2; 1** **–** **3**
**Mean ± SD**					
**hoa**	**0.4**	**1.04**	**1.39**	<0.001 **	**1** **–** **2; 1** **–** **3**
**Median (Min-Max)**	**(0.21–0.64)**	**(0.33–3.35)**	**(0.41–2.94)**		
**IQR (Q1–Q3)**	**(0.32–0.50)**	**(0.74–1.73)**	**(0.96–1.90)**		
**coma**	**0.23**	**0.86**	**1.18**	<0.001 **	**1** **–** **2; 1** **–** **3**
**Median (Min-Max)**	**(0.06–0.53)**	**(0.22–3.05)**	**(0.32–2.49)**		
**IQR (Q1–Q3)**	**(0.16–0.33)**	**(0.55–1.57)**	**(0.77–1.60)**		
sa	0.21	0.17	0.18	0.491 **	
Median (Min-Max)	(0.07–12.04)	(0.02–0.68)	(0–1.12)		
IQR (Q1–Q3)	(0.14–0.27)	(0.11–0.26)	(0.09–0.25)		

Values are presented as mean ± standard deviation (SD) for normally distributed variables or as median (minimum–maximum) and interquartile range (IQR, Q1–Q3) for non-normally distributed variables. ^1^ = Control group, ^2^ = Symmetric KC group, ^3^ = Asymmetric KC group. *p*-values calculated using the Kruskal–Wallis test (**) or one-way ANOVA (*), with post hoc Bonferroni correction where applicable. * ANOVA test, ** Kruskal–Wallis H test, bold: statistically significant, KC: Keratoconus, oaRI: Ophthalmic Artery Resistive Index, oaPI: Ophthalmic Artery Pulsatility Index, craRI: Central Retinal Artery Resistive Index, craPI: Central Retinal Artery Pulsatility Index, pcaRI: Short Posterior Ciliary Artery Resistive Index, pcaPI: Short Posterior Ciliary Artery Pulsatility Index, Kvf: Anterior vertex refractive index, Kvb: Posterior vertex refractive indices index, MCCT: Minimum central corneal thickness, CV: Corneal volume, SimK1, SimK2 = Simulated Keratometry Readings (D), hoa, coma, sa: higher-order aberrations, and SD: Standart deviation.

**Table 5:** Distribution of Blood Flow and Topographic Measurements in the Eye without KC in Asymmetric Patients Compared to Symmetric and Control Groups.

Variables	Control ^1^	Symmetric ^2^	Asymmetric No KC ^3^	*p*-Value	Post-HOC
**oaRI**	**0.78**	**0.78**	**0.74**	0.030 **	**1** **–** **3; 2** **–** **3**
**Median (Min–Max)**	**(0.64–0.89)**	**(0.61–0.87)**	**(0.6–9.6)**		
**IQR (Q1–Q3)**	**(0.71–0.82)**	**(0.66–0.82)**	**(0.66–1.02)**		
**oaPI**	**1.8**	**2**	**1.62**	0.028 **	**2** **–** **3**
**Median (Min–Max)**	**(1.02–3.14)**	**(1.26–2.77)**	**(1.02–3.34)**		
**IQR (Q1–Q3)**	**(1.50–2.02)**	**(1.58–2.26)**	**(1.32–2.01)**		
**craRI**	0.72 ± 0.08	0.74 ± 0.07	0.74 ± 0.09	0.320 *	–
**(Mean ± SD)**					
**craPI**	1.41	1.6	1.38	0.080 **	–
**Median (Min–Max)**	(0.74–2.74)	(1.07–2.88)	(0.68–2.73)		
**IQR (Q1–Q3)**	(1.20–1.57)	(1.33–1.89)	(1.14–1.93)		
**pcaRI**	0.72	0.74	0.7	0.467 **	–
**Median (Min–Max)**	(0.58–0.83)	(0.56–1)	(0.52–6.4)		
**IQR (Q1–Q3)**	(0.67–0.76)	(0.68–0.78)	(0.65–0.76)		
**pcaPI**	1.34	1.48	1.27	0.239 **	–
**Median (Min–Max)**	(0.91–2.41)	(0.89–5.05)	(0.75–2.55)		
**IQR (Q1–Q3)**	(1.19–1.64)	(1.24–1.71)	(1.13–1.60)		
**Kvf**	**4**	**25**	**9**	<0.001 **	**All**
**Median (Min–Max)**	**(1.5–7)**	**(5.5–81.5)**	**(3–42)**		
**IQR (Q1–Q3)**	**(3.50–5.25)**	**(16.0–31.0)**	**(5.0–14.0)**		
**Kvb**	**10**	**59**	**25**	<0.001 **	**All**
**Median (Min–Max)**	**(6–16.5)**	**(9–168)**	**(7–102)**		
**IQR (Q1–Q3)**	**(8.50–11.75)**	**(35.5–73.0)**	**(14.0–33.0)**		
**MCCT**	**531.95 ± 29.36**	**459.96 ± 47.04**	**492.97 ± 34.54**	<0.001 *	**All**
**(Mean ± SD)**					
**CV**	**0.04**	**2.46**	**0.88**	<0.001 **	**All**
**Median (Min–Max)**	**(0–0.37)**	**(0–8.61)**	**(0.12–3.46)**		
**IQR (Q1–Q3)**	**(0.00–0.12)**	**(1.60–3.72)**	**(0.51–1.34)**		
**CylD**	**−0.64**	**−2.33**	**−1.28**	<0.001 **	**All**
**Median (Min–Max)**	**(–1.38–−0.13)**	**(–7.42–−0.4)**	**(–4.98–−0.2)**		
**IQR (Q1–Q3)**	**(–0.87–−0.39)**	**(–3.21–−1.73)**	**(–1.85–−0.69)**		
**SimK1**	**42.95 ± 1.53**	**44.72 ± 2.27**	**43.69 ± 2.07**	<0.001 *	**1** **–** **2**
**(Mean ± SD)**					
**SimK2**	**43.76 ± 1.58**	**47.1 ± 2.63**	**45.26 ± 2.41**	<0.001 *	**All**
**(Mean ± SD)**					
**hoa**	**0.4**	**1.04**	**0.49**	<0.001 **	**1** **–** **2; 2** **–** **3**
**Median (Min–Max)**	**(0.21–0.64)**	**(0.33–3.35)**	**(0.15–2.41)**		
**IQR (Q1–Q3)**	**(0.32–0.50)**	**(0.74–1.73)**	**(0.32–0.69)**		
**Coma**	**0.23**	**0.86**	**0.36**	<0.001 **	**1** **–** **2; 2** **–** **3**
**Median (Min–Max)**	**(0.06–0.53)**	**(0.22–3.05)**	**(0.04–1.75)**		
**IQR (Q1–Q3)**	**(0.16–0.33)**	**(0.55–1.57)**	**(0.12–0.49)**		
**sa**	**0.21**	**0.17**	**0.08**	<0.001 **	**1** **–** **3; 2** **–** **3**
**Median (Min–Max)**	**(0.07–12.04)**	**(0.02–0.68)**	**(0–1.04)**		
**IQR (Q1–Q3)**	**(0.14–0.27)**	**(0.11–0.26)**	**(0.03–0.15)**		

Values are presented as mean ± standard deviation (SD) for normally distributed var-iables or as median (minimum–maximum) and interquartile range (IQR, Q1–Q3) for non-normally distributed vari-ables. ^1^ = Control group, ^2^ = Symmetric KC group, ^3^ = Asymmetric KC group. *p*-values calculated using the Krus-kal–Wallis test (**) or one-way ANOVA (*), with post hoc Bonferroni correction where applicable. * ANOVA test, ** Kruskal–Wallis H-Test, bold: statistically significant KC: Keratoconus, oaRI: Ophthalmic Artery Resistive Index, oaPI: Ophthalmic Artery Pulsatility Index, craRI: Central Retinal Artery Resistive Index, craPI: Central Retinal Artery Pulsatility Index, pcaRI: Short Posterior Ciliary Artery Resistive Index, pcaPI: Short Posterior Ciliary Artery Pulsatility Index, Kvf: Anterior vertex refractive index, Kvb: Posterior vertex refractive indices index, MCCT: Minimum central corneal thickness, CV: Corneal volume, SimK1, SimK2 = Simulated Keratometry Readings (D), hoa, coma, sa: higher-order aberrations, and SD: Standart deviation.

## Data Availability

The original contributions presented in the study are included in the article, further inquiries can be directed to the corresponding authors.
